# Supplementary material for: Evidence of the Autophagic Process during the Fish Immune Response of Skeletal Muscle Cells against Piscirickettsia salmonis
Source: Animals (Basel). 2023 Feb 28;13(5):880. doi: 10.3390/ani13050880 (PMC10000225; doi:10.3390/ani13050880)
Supplement: Supplementary file 1 [file animals-13-00880-s001.zip › animals-2212057-figure S4.pdf]

LC3-II WB

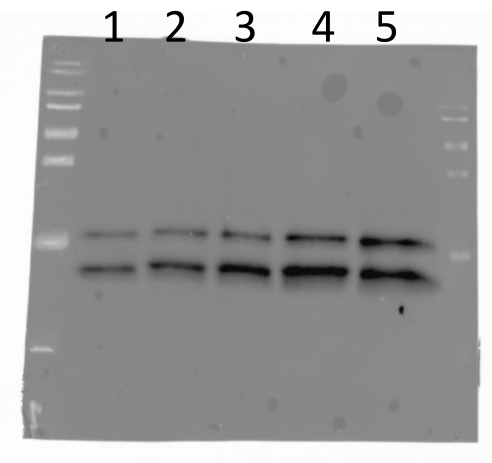

Assay 1
1. Control
2. P. salmonis induced
3. Rapamycin treated
4. *P. salmonis* induced
5. Rapamycin treated

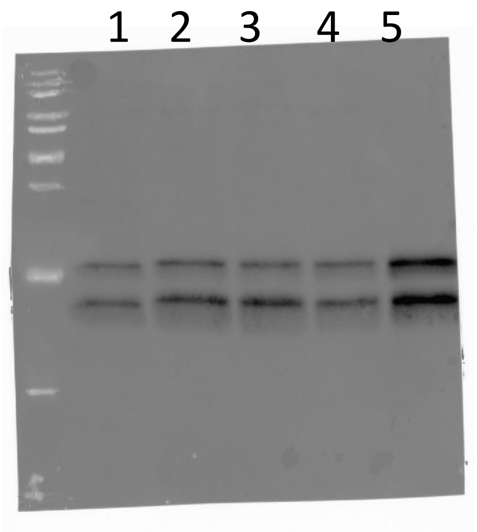

Assay 2
1. Control
2. P. salmonis induced
3. Rapamycin treated
4. *P. salmonis* induced
5. Rapamycin treated

Actin WB

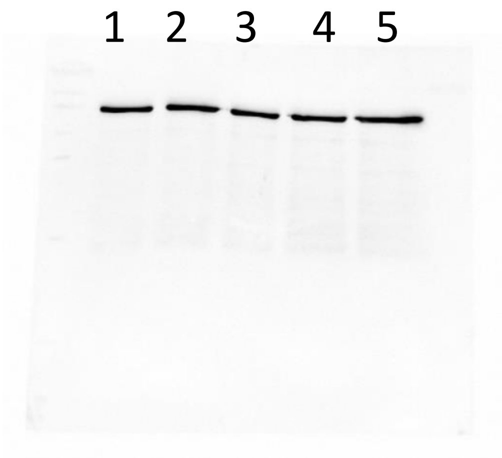

1. Control
2. P. salmonis induced
3. Rapamycin treated
4. *P. salmonis* induced
5. Rapamycin treated

Rab7 WB

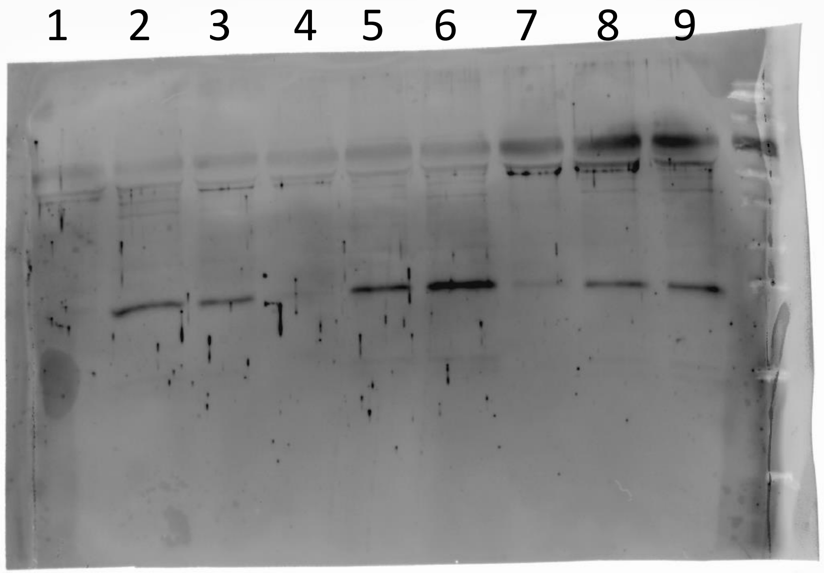

1. Rapamycin treated
2. Rapamycin treated
3. Rapamycin treated
4. *P. salmonis* induced
5. *P. salmonis* induced
6. *P. salmonis* induced
7. Control
8. Control
9. Control

Actin WB

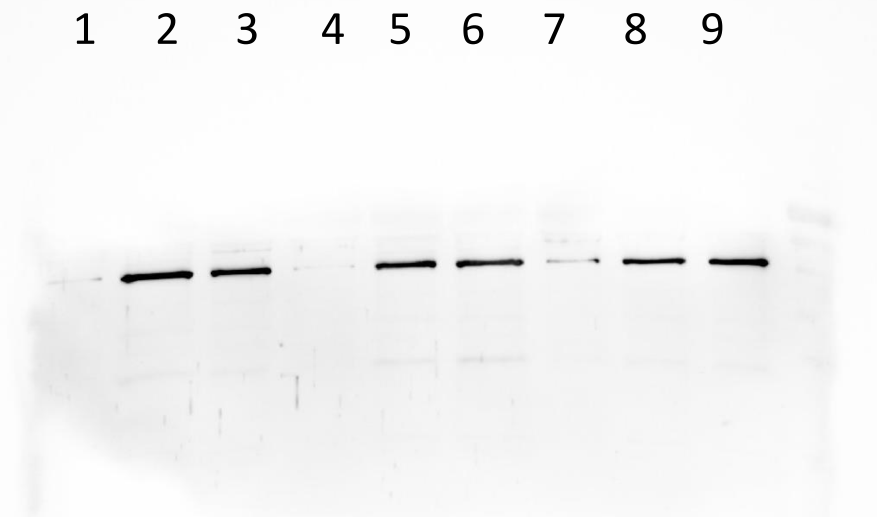

1. Rapamycin treated
2. Rapamycin treated
3. Rapamycin treated
4. *P. salmonis* induced
5. *P. salmonis* induced
6. *P. salmonis* induced
7. Control
8. Control
9. Control
