# Supplementary material for: Evidence of the Autophagic Process during the Fish Immune Response of Skeletal Muscle Cells against Piscirickettsia salmonis
Source: Animals (Basel). 2023 Feb 28;13(5):880. doi: 10.3390/ani13050880 (PMC10000225; doi:10.3390/ani13050880)
Supplement: Supplementary file 1 [file animals-13-00880-s001.zip › Table S1, primers for qPCR.pdf]

Supplementary Table S1. Primer used for qPCR analysis

Gene	Sequence 5' - 3'		TM (°C)	% E	GenBank code/reference
tnfα	Forward	GCAGCCATCCATTTAGAGGGTGAA	60	100	DQ787157.1
	Reverse	CTAAACGAAGCCTGGCTGTAAACG			
tlr3	Forward	CTGATTGCTTGAAGCCCATT	60	99.5	NM_001124578.1
	Reverse	GCCTTTGAAGGTGGTGTTGT			
tlr9	Forward	GGACAACCTGGCGTACCTTA	60	102.38	NM_001129991.2
	Reverse	CCCATGCCTCTCATTAGGAA			
il-1β	Forward	CCCCATTGAGACTAAAGCCA	60	109.8	XM_014170479.2
	Reverse	GCAACCTCCTCTAGGTGCAG			
il-8	Forward	GCCCTCCTGACCATTACTGA	60	101.83	NM_001140710.3
	Reverse	AAATCTCCTGACCGCTGTTG			
hepcidin	Forward	GAAGGCCTTTAGTGTTGCAGTGGT	60	93	AF542965.1
	Reverse	GTTGATGTTCCCCAACTGGACTGT			
mhc-II	Forward	AATCAGAGTGACCTGGTTGAG	60	110	AJ439070.1
	Reverse	GTGGGAGAGGATCTGGTAGTA			
mhc-I	Forward	CAACGCCACAGGCAGTCA	60	97	JN561338.1
	Reverse	CGGTACTCATTCTGAGCTGTGTTAC			
becn1	Forward	TGGATGTCGAGAAGGGCAA	60	104.6	[15]
	Reverse	ATGAACTTGAGCGCCTTGG			
atg9	Forward	TGGGCAATCTGGTGTTCCTC	60	99.12	[15]
	Reverse	TGCACGTTTGTACTTGGGGT			
atg12	Forward	CTTCAACCCCACAACAGCCTA	60	102.4	[15]
	Reverse	TCCTCCCTTTCTCTACCGACC			
gabarap	Forward	AGCCCCCAAAGCAAGGATAG	60	92.37	[15]
	Reverse	CGCAGGTGGATTCGTTTTCG			
atg5	Forward	ATTTCCCAGAGCGTGACCTG	60	94.43	[15]
	Reverse	TGTCGTTGATGACCTGGCTC			
atg4	Forward	GCTGCGATGTGGACAGATGAT	43	95.43	[15]
	Reverse	AAGGAAGGCGTTGAGGATACC			
lc3	Forward	CAGCACCCCAACAAGATCCC	60	95.23	[15]
	Reverse	GCGCCTCCTGATGATTTTGA			
rab5a	Forward	ACGACATCACAAACGAGGAG	60	99.14	XM_014178400.2
	Reverse	CCTGACAGAGCGATGACAAT			
rab5c	Forward	TGACATCACCAACACAGATACA	60	92.46	XM_014192873.2
	Reverse	TTCCCTGCCAGTGCAATAA			
rab7a	Forward	TAAGAAGGTGCTGCTGAAGG	60	93.30	XM_014128290.2
	Reverse	GGCCTTGTACTGGTTACTGAA			
rab11a	Forward	TGGGAACCAGAGATGATGAATAC	60	96.05	XM_014127757.2
	Reverse	GTGAAACGAGAGAGCAGGTT			
beta actin	Forward	CCCATCTACGAGGGTTACGC	62	100.5	XM_014194536.2
	Reverse	ACTCCTGCTTGCTGATCCAC			
ef-1α	Forward	CCCCTCCAGGACGTTTACAAA	56	104.6%	NM001123629.1
	Reverse	CACACGGCCCACAGGTACA			
