# Supplementary figures and images for: Evidence of the Autophagic Process during the Fish Immune Response of Skeletal Muscle Cells against Piscirickettsia salmonis
Source: Animals (Basel). 2023 Feb 28;13(5):880. doi: 10.3390/ani13050880 (PMC10000225; doi:10.3390/ani13050880)

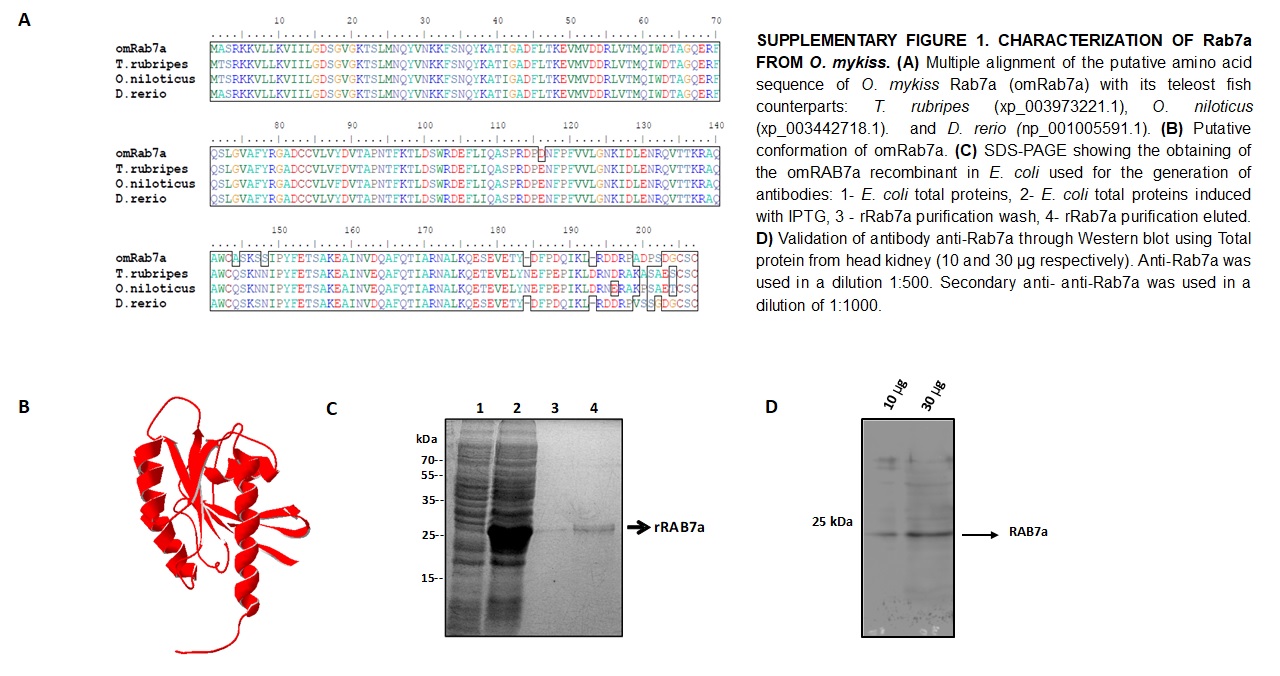

Supplement: Supplementary file 1 [file animals-13-00880-s001.zip › Figure S1.jpg]

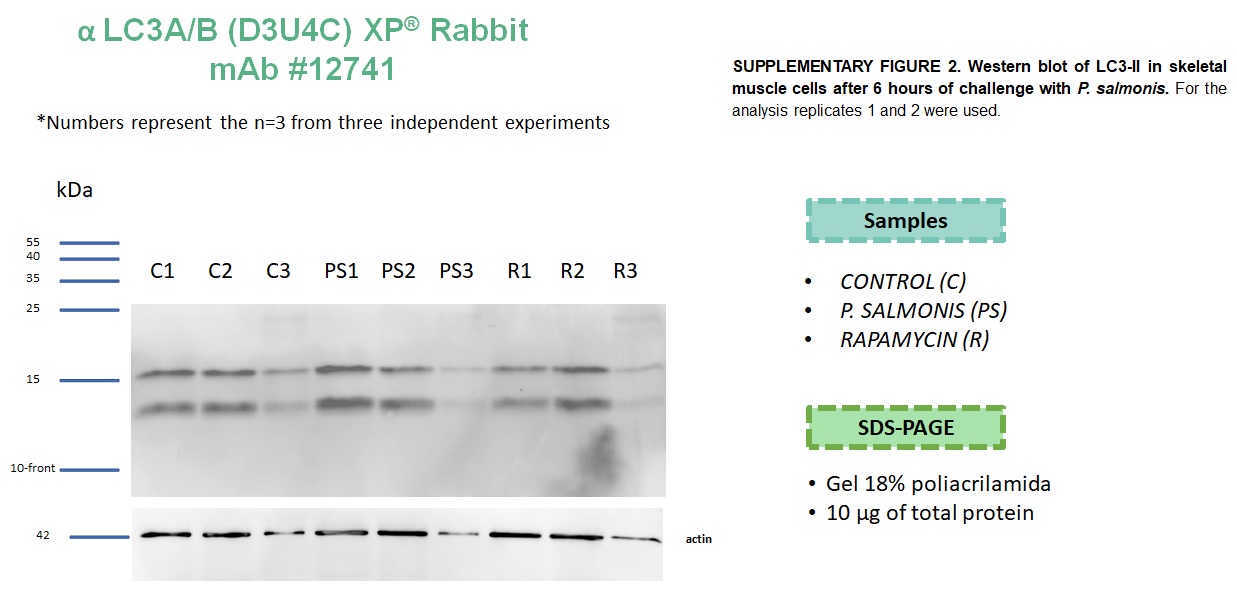

Supplement: Supplementary file 1 [file animals-13-00880-s001.zip › Figure S2.jpg]

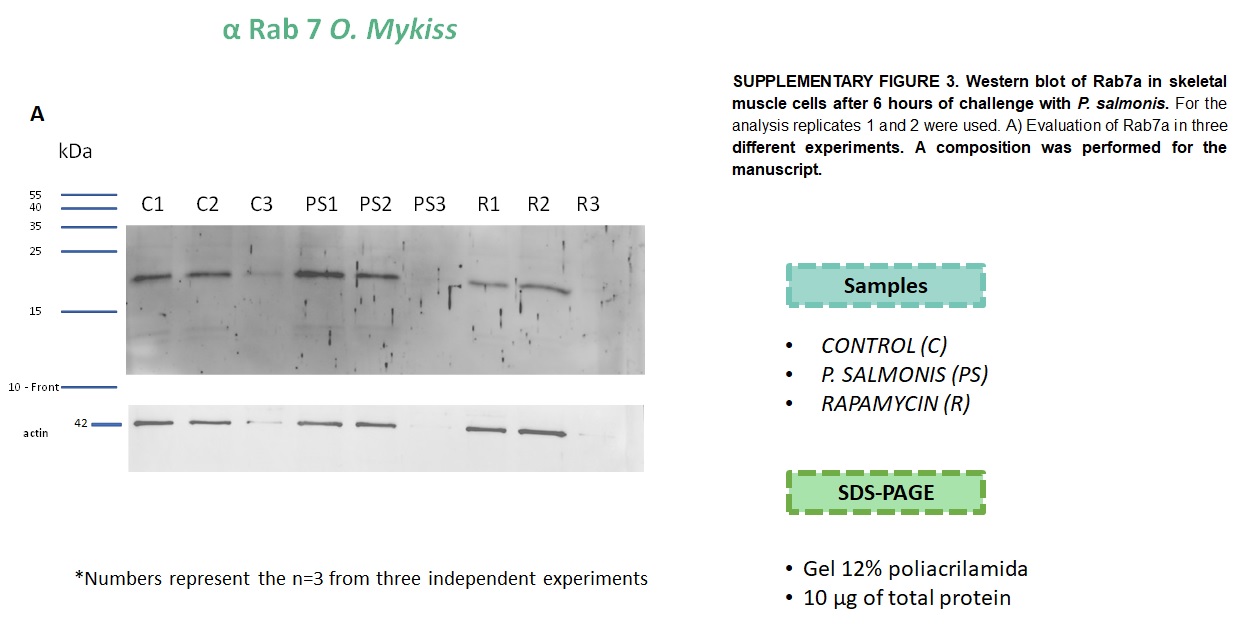

Supplement: Supplementary file 1 [file animals-13-00880-s001.zip › Figure S3.jpg]
